# Fact or Factitious? A Psychobiological Study of Authentic and Simulated Dissociative Identity States

**DOI:** 10.1371/journal.pone.0039279

**Published:** 2012-06-29

**Authors:** A. A. T. Simone Reinders, Antoon T. M. Willemsen, Herry P. J. Vos, Johan A. den Boer, Ellert R. S. Nijenhuis

**Affiliations:** 1 King’s College London, Institute of Psychiatry, Department of Psychosis Studies, London, United Kingdom; 2 Department of Neuroscience, University Medical Center Groningen, and BCN Neuroimaging Center, University of Groningen, Groningen, The Netherlands; 3 Department of Nuclear Medicine & Molecular Imaging, University Medical Center Groningen, University of Groningen, The Netherlands; 4 Outpatient Department Addiction Clinic Groningen/Drenthe, The Netherlands; 5 University Center of Psychiatry, University Medical Center Groningen, University of Groningen, The Netherlands; 6 Top Referent Trauma Center Mental Health Care Drenthe, Assen, The Netherlands; Federal University of Rio de Janeiro, Brazil

## Abstract

**Background:**

Dissociative identity disorder (DID) is a disputed psychiatric disorder. Research findings and clinical observations suggest that DID involves an authentic mental disorder related to factors such as traumatization and disrupted attachment. A competing view indicates that DID is due to fantasy proneness, suggestibility, suggestion, and role-playing. Here we examine whether dissociative identity state-dependent psychobiological features in DID can be induced in high or low fantasy prone individuals by instructed and motivated role-playing, and suggestion.

**Methodology/Principal Findings:**

DID patients, high fantasy prone and low fantasy prone controls were studied in two different types of identity states (neutral and trauma-related) in an autobiographical memory script-driven (neutral or trauma-related) imagery paradigm. The controls were instructed to enact the two DID identity states. Twenty-nine subjects participated in the study: 11 patients with DID, 10 high fantasy prone DID simulating controls, and 8 low fantasy prone DID simulating controls. Autonomic and subjective reactions were obtained. Differences in psychophysiological and neural activation patterns were found between the DID patients and both high and low fantasy prone controls. That is, the identity states in DID were not convincingly enacted by DID simulating controls. Thus, important differences regarding regional cerebral bloodflow and psychophysiological responses for different types of identity states in patients with DID were upheld after controlling for DID simulation.

**Conclusions/Significance:**

The findings are at odds with the idea that differences among different types of dissociative identity states in DID can be explained by high fantasy proneness, motivated role-enactment, and suggestion. They indicate that DID does not have a sociocultural (e.g., iatrogenic) origin.

## Introduction

Despite its inclusion in the Diagnostic Manual for Mental Disorders [1], the genuineness of dissociative identity disorder (DID) continues to be disputed. Supporters of the diametrically opposed trauma-related and non-trauma-related views have been engaged since decades in a passionate debate regarding its validity as a mental disorder, and whether it is related to traumatization or to fantasy proneness, suggestibility, suggestion, and simulation [2]–[10].

The non-trauma-related position [2], [3], [7], [11]–[13], also referred to as the sociocognitive model of DID [14]–[16], holds that DID is a simulation caused by high suggestibility and/or fantasy proneness [17]–[21], suggestive psychotherapy and other suggestive sociocultural influences (e.g., the media and/or the church). According to this model, “[t]he rules for enacting the [DID] role […] are as follows: (a) Behave as if you are two (or more) separate people who inhabit the same body. (b) Act as if the you I have been addressing thus far is one of those people and as if the you I have been talking to is unaware of the other coinhabitants. (c) When I provide a signal for contacting another coinhabitant, act as though you are another person. To the extent that patients behave in terms of these rules, the “classic” symptoms [of DID] follow by implication and do not have to be taught through direct instruction or further suggestion”, Spanos (p.239 [15]). Fantasy proneness and suggestibility are highly correlated [18], [22]–[25], and dissociative symptoms were found to be correlated with fantasy proneness, heightened suggestibility, and susceptibility to pseudomemories [11], [26].

To date, the position that DID is caused by sociocultural factors and personal features such as fantasy proneness has not been tested in studies involving DID patients, and evidence that the complex phenomenology and psychobiology of DID can be created and sustained over time by these factors is lacking [27]–[30]. Despite this lack of empirical support, the sociocognitive and fantasy based model of DID is influential in contemporary psychiatry and there have been proposals to prevent the inclusion of DID in the DSM-V [31].

The trauma-related perspective entails that DID is related to a combination of factors that include chronic emotional neglect and emotional, physical, and/or sexual abuse from early childhood, insufficient integrative capacity, attachment disorder, and lack of affect-regulation by caretakers [27], [32]–[35]. In this view DID is thought to be at the far end of the spectrum of trauma-related psychiatric disorders, i.e. being a severe form of post-traumatic stress disorder (PTSD) [33], [36].

Holders of the trauma-related view acknowledge that: some features of dissociative identity states can be influenced by sociocultural factors [33], that false positive cases of DID have evolved in a treatment setting, and that some psychiatric patients imitate DID [37]. However, they also note that there are differences between authentic and imitated DID and that there is no evidence that DID can (sub-)consciously be created by sociocultural factors [27]. Furthermore, even if DID symptoms can be created iatrogenically or enacted [14] this does not mean that genuine trauma-related DID does not exist [38].

According to the DSM-IV [1], DID is characterized by, among others, the presence of two or more distinct `identities’ or `personality states’. Different proposed labels include `different emotional states’, `alters’, `dissociative parts of the personality’ [33], and `dissociative identity states.’ Following previously used descriptions and terminology [39], [40] different types of dissociative identity states are indicated here as neutral identity states (NIS) and trauma-related identity states (TIS). These indicators are derived from the terms ‘apparently normal part of the personality (ANP)’ and ‘emotional part of the personality (EP)’ respectively, which are used in the theory of structural dissociation [33], [41]. This theory defines dissociation as a division of personality into different types of subsystems, each with their own first-person perspective, that is, their own point of view as to who they are, what the world is like, and how they relate to that world [42]. As NIS DID patients concentrate on functioning in daily life, commonly try to hide their pathology, and have not sufficiently integrated (e.g., have partial or complete amnesia) traumatic memories. That is, NIS fails to relate the trauma-related nature to its self [39]. In contrast, TIS does have conscious access to these memories, recalls them as personal experiences and is bodily and emotionally affected by them. That is, as TIS the patients are fixated in traumatic memories and engage in defensive actions such as freeze and flight, when they are or feel threatened [41], [43], thereby activating fast subcortical response routes in the brain [40], [44]. TIS who engage in active kinds of physical defence (e.g., freeze, flight, fight) would involve dominance of the sympathetic nervous system, whereas those who engage in total submission (i.e.,playing dead) would be primarily mediated by the dorsal vagal branch of the parasympathetic nervous system [45].

Proponents of the sociocognitive view have argued that the different patterns of subjective, psychophysiological, and neural activity for NIS and TIS in response to a trauma-memory script that Reinders et al. [39], [40] documented, might be due to fantasy proneness, suggestion and role-playing, and that they do not prove a traumagenic origin of DID. Obtaining independent proof of childhood traumatization in adulthood is most difficult. However, the claim that the previously reported results constitute effects of fantasy proneness, suggestion, and role-playing is open to test. Thus, the present study involves a psychobiological comparison between NIS and TIS engaging in active kinds of physical defence in DID patients (i.e., the DID identity states from Reinders et al. [39], [40]), and simulated NIS and TIS in high and low fantasy prone mentally healthy women who do not report a trauma history and who are instructed and motivated to role-play these different identity states (i.e., simulated identity states).

The *a priori* hypotheses of the current study were: (i) important previously found psychophysiological and neurobiological differences between NIS and TIS engaging in active kinds of physical defence in DID patients [39], [40] are upheld when controlling for fantasy proneness, suggestion, and instructed and motivated role-playing, and (ii) the upheld psychophysiological and neurobiological differences for NIS and TIS in DID patients include higher sympathetic nervous system activation (e.g. higher heart rate and systolic bloodpressure) and subcortical activity (e.g. the amygdala and caudate nucleus) for TIS in DID, and (iii) hyperactivation of the cortical multimodal posterior association areas (e.g. the intraparietal sulcus and (pre-)cuneus) for NIS in DID when listening to personal trauma scripts.

## Results

Twenty-nine subjects participated in the brain imaging study: 11 patients with dissociative identity disorder (DID), 10 high fantasy prone DID simulating controls, and 8 low fantasy prone DID simulating controls. The controls were instructed to enact the two DID identity states: a neutral identity state (NIS) and a trauma-related identity state (TIS). Brain imaging data, autonomic (systolic and diastolic blood pressure, discrete heart rate and heart rate variability (HRV)) and subjective (controls’ subjective sensorimotor and emotional experiences) reactions were obtained. DID patients, as well as high fantasy prone and low fantasy prone controls were studied in the two different types of identity states during a memory script (MS) driven (neutral or trauma-related autobiographical texts) imagery paradigm. The brain imaging data of the three groups was statistically analyzed in SPM5 in a three-by-two-by-two factorial design which allows for the assessment of various effects, e.g., main effects and simple subtraction analyses (within and between identity state) within and between the three groups.

### Autonomic and Subjective Reactions

**Figure 1:**
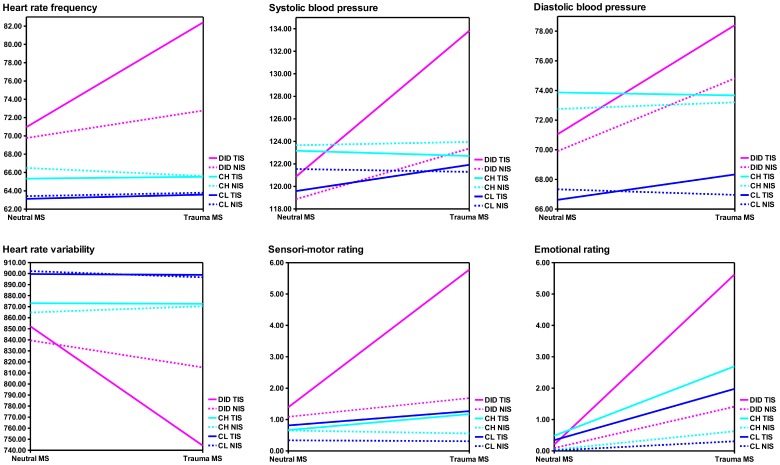
Graphical representation of averages and direction of subjective emotional experiences, subjective sensori-motor experiences, and cardiovascular responses. The dashed line depicts the response of the neutral identity state (NIS) when listening to the neutral or trauma-related memory script (MS). The solid line depicts the response of the traumatic identity state (TIS) when listening to the neutral or trauma related MS. All three groups are presented per variable: the dissociative identity disorder patients (DID) in pink, the high fantasy prone DID simulating controls (CH) in cyan and the low fantasy prone DID simulating controls (CL) in blue. See Table 1 for the statistical values.

Statistical results of the autonomic and subjective reactions analyses between the three groups are presented in Table 1. Mean values and the direction of the responses are depicted in Figure 1. Significant differences were found for most of the measured variables between the DID patients and both control groups (see for details Table 1) for dissociative identity state (DIS), DIS*group, MS, MS*group, DIS*MS, and DIS*MS*group.

**Table 1:** Between group: Subjective and autonomic reactions.

	*Between group: DID versus CH*						*Between group: DID versus CL*					
	DIS	DIS* Group	MS	MS* Group	DIS *MS	DIS*MS*Group	DIS	DIS*Group	MS	MS* Group	DIS *MS	DIS*MS* Group
**Subjective ratings**												
sensory rating	<0.001**	<0.001**	<0.001**	<0.001**	<0.001**	<0.001**	<0.001**	<0.007**	<0.001**	<0.001**	<0.001**	0.001**
emotional rating	<0.001**	n.s.	<0.001**	0.001**	<0.001**	0.031*	<0.001**	0.076	<0.001**	<0.001**	<0.001**	0.030*
**Autonomic reactions**												
heart rate frequency	0.011*	0.002**	<0.001**	<0.001**	0.009*	0.036*	0.018*	0.010*	<0.001**	<0.001**	0.021*	0.023*
systolic blood pressure	0.058	0.015*	0.006**	0.005**	0.044*	0.018*	0.080	0.034*	0.006**	0.025*	0.017*	n.s.
diastolic blood pressure	0.043*	n.s.	0.001**	0.002**	n.s.	n.s.	n.s.	n.s.	0.001**	0.004**	n.s.	n.s.
HRV-AVG	n.s.	0.078	0.006**	0.003**	0.017*	0.036*	n.s.	n.s.	0.009*	0.015*	0.054	0.033*

Factorial statistical analyses of the between group (DID versus high or low fantasy prone DID simulating healthy controls, respectively) subjective reactions (emotional and sensori-motor ratings) and autonomic (discrete heart rate, systolic and diastolic blood pressure and heart rate variability) measurements. The statistical analyses consist of the two main effects and the accompanying interaction effect. Statistical values are reported in *p* values.

** =  *p*<0.0083 (i.e., corrected for multiple comparisons).

* =  *p*<0.05 (i.e., uncorrected for multiple comparisons).

DID  =  dissociative identity disorder.

CH  =  high fantasy prone DID simulating controls.

CL  =  low fantasy prone DID simulating controls.

DIS  =  dissociative identity state.

DIS*Group  =  interaction effect.

MS  =  memory script.

MS*Group  =  interaction effect.

DIS * MS  =  interaction effect.

DIS*MS*Group  =  interaction effect.

HRV-AVG  =  average of normal-to-normal time intervals.

### Regional Cerebral Blood Flow Changes

#### Covariate data

T-tests were used to test if a significant (*p*<0.05) difference in regional cerebral bloodflow (rCBF) variance between the DID and control groups was explained by the subjective or objective covariates (i.e. the principal components (PC), see below). No brain areas for which a significant difference was present between the DID patients and the high or low fantasy prone controls respectively were found.

**Table 2:** Main effects.

			*Within group*					*Between group*									
			DID only					DID - CH					DID - CL				
L/R	Brain region I = *a priori*	BA	x	y	z	T	kE	x	y	z	T	kE	x	y	z	T	kE
**Main Effect TIS**																	
*Cortical areas*																	
L	Insula	BA 13 IIIb	−46	−20	14	4.76	258 1						−46	−24	18	4.41**	302 1
R	Insula	BA 13 IIIb	32	−24	18	3.87	42 2						46	−2	22	3.98**	1201 3
L	Orbitofrontal cortex	BA 11 IV						−30	34	−22	3.95	96					
		BA 11 IV						−24	56	−20	3.73	61					
L	Parietal operculum I	BA 40 IIIb	−54	−14	16	4.02	258 2						−54	−14	16	3.45*	302 2
R	Parietal operculum I	BA 40/43 II	52	2	20	3.97	64 1										
		BA 40/43 II	48	−6	26	3.52	64 2										
R	Postcentral gyrus I	BA 43 III	68	−14	14	4.60	177 1	68	-10	16	4.25*	94 1	68	−14	18	4.65**	146 1
R	Precentral gyrus I	BA 6 IIIb	66	6	20	4.06	177 2						66	8	20	3.57**	146 2
L	S. Temporal gyrus	BA 22/42 IV											−68	−28	18	3.63**	64 1
		BA 22/42 IV											−60	−34	16	3.20	64 2
R	S. Temporal gyrus	BA 22/42 IV						70	−22	16	3.27	94 2					
		BA 22/42 IV						72	−40	10	3.52	17					
*Subcortical areas*																	
L	Amygdala I	IIIa	−6	−6	−26	4.30	29	−6	−6	−26	3.73*	35					
L	Caudate nucleus (anterior part)	IV											−24	14	18	3.53**	338 3
R	Caudate nucleus (caudal part) I	II	20	−26	20	4.56	42 1										
L	Caudate nucleus (dorsal part) I	III	−26	−10	20	4.72	203 2	−24	−4	20	3.88*	144 2	−22	−6	20	4.90**	338 1
		III	−18	−4	20	4.98	203^1^	−12	2	18	3.96*	144 1	−14	4	18	4.76**	338 2
R	Caudate nucleus (dorsal part) I	III	22	2	22	4.69	229 1	24	4	20	4.84*	625 1	26	−4	16	5.73**	1201 1
		IV						24	−4	18	4.71*	625 2					
R	Caudate nucleus(lateral-dorsal part) I	IIIb	32	−2	22	4.05	229 2						32	6	20	4.74**	1201 2
L	Caudate nucleus (tail) I	II	−26	−36	12	4.45	43										
R	Putamen I	IV						26	−16	16	3.90*	625 3					
*Cerebellum*																	
L	Cerebellar Tonsil (nodule) I	IIIa	−6	−44	−34	4.73	88	−6	−46	−34	3.60*	29					
R	Nucleus Dentatus I	IV						12	−48	−30	3.48*	10					
**Main Effect NIS**																	
*Cortical areas*																	
R	Angular gyrus	BA 39 IIIa	42	−72	34	4.16**	508 2	44	−62	30	3.66	238 2					
		BA 39 IV						50	−66	34	3.41	238 3					
R	Anterior Cingulate gyrus I	BA 32 II	2	46	6	4.94**	1039 1										
R	Cingulate gyrus I	BA 32 IIIa	2	18	42	4.93**	2140 3	4	8	50	3.24	10					
		BA 32 IV						8	16	34	3.44*	18					
L	Cingulate sulcus	BA 31 II	−14	−40	40	3.91**	419 3										
R	Cingulate sulcus I,u	BA 6/24 IIIb	16	−12	44	5.38**	2140 2						18	−12	46	3.33	10
L	Cuneus I	BA 18/19 IIIa	−16	-88	32	3.82**	292 3	−16	-88	32	3.79*	577 2					
		BA 18/19 IIIa	−10	−78	20	3.91**	292 2	−10	−78	20	3.61*	577 3					
R	Cuneus I	BA 18/19 IIIa	14	−78	34	3.87**	102 2	12	−80	22	3.75*	184 1					
		BA 18/19 IV						16	−90	38	3.62*	184 2					
X	Cuneus	BA 18 II	0	−90	40	3.77**	13										
R	Fusiform gyrus I	BA 19 II	42	−64	−20	5.00**	259 1										
		BA 19/37 II	28	−58	−14	3.60**	259 2										
L	I. Frontal gyrus	BA 47 IV											−42	28	−12	3.67	42
R	I. Frontal gyrus	BA 45/47 II	46	32	2	3.58**	11										
L	Medial Frontal gyrus	BA 10 IV											−4	66	2	3.38	41
L	M. Frontal gyrus	BA 10 II	−20	66	8	3.52**	9										
R	S./Medial Frontal gyrus	BA 10 IIIb	12	64	4	4.94**	1039 2						14	64	4	3.27	11
		BA 10 II	12	66	12	4.75**	1039 3										
R	S. Frontal gyrus I	BA 8 IIIb	12	38	56	4.81**	156 1						12	36	58	3.51	9
		BA 6 II	14	26	66	3.38**	156 2										
L	S. Frontal sulcus I	BA 6 IIIb	−32	−2	48	5.47**	2140 1						−34	−4	50	3.60*	66 1
		BA 6 IV											−40	4	52	3.36*	66 2
R	S. Frontal sulcus I	BA 4/6 IV						26	−14	52	3.27*	26 2					
		BA 4/6 IV						28	−16	44	3.61*	26 1					
L	Lingual gyrus	BA 18 IV											−10	−82	−10	3.23	64 2
		BA 18 IV											−8	−90	−10	3.50	64 1
R	Lingual gyrus I	BA 18 III	16	−82	−14	4.31**	286 1	14	−80	−8	3.60	35	14	−76	−12	3.44*	32
		BA 18 II	20	−72	−18	3.67**	286 2										
		BA 18 II	22	−74	−8	3.58**	286 3										
L	M. Occipital gyrus I	BA 18 II	−28	−88	14	4.15**	79 1										
		BA 18 II	−24	−98	16	3.74**	79 2										
L	S. Occipital gyrus/Angular gyrus I	BA 19/39 III	−38	−80	28	6.12**	299	−42	−80	32	3.68*	51	−40	-80	32	4.03*	89
R	S. Occipital sulcus I	BA 19 II	30	−86	32	3.87**	508 3										
	S. Occipital sulcus I/Cuneus I	BA 18/19 II	18	−88	40	4.03**	102 1										
R	Occipitotemporal sulcus I	BA 20/37 III	48	−40	−12	4.66**	311 1	48	−40	−12	4.68*	187 1	44	−36	−12	4.43**	134
		BA 20/37 IV						46	-52	-6	3.74*	187 2					
L	Parahippocampal gyrus I	BA 35 IV											−38	−44	-8	4.41**	142 1
		BA 35 IV											−38	−42	−16	3.57*	142 2
		BA 35 IV											−26	−22	−14	3.34	10
		BA 35 IIIb	−14	−46	−2	3.49**	191 2						−8	−38	−10	5.06**	426
R	Parahippocampal gyrus I	BA 35/36 II	22	−44	−2	4.26**	185 1										
		BA 35/36 II	24	−34	−6	4.10**	185 3										
		BA 35/36 II	28	−42	−8	4.16**	185 2										
L	Intra-Parietal sulcus I	BA 7/40 IIIa	−34	−50	36	4.59**	419 2	−34	−48	36	3.90*	95					
		BA 7/40 II	−26	−48	40	5.50**	419 1										
R	Intra-Parietal sulcus I	BA 7/40 IIIa	24	−38	44	5.53**	390 1	24	-40	44	4.80*	316 1					
		BA 7/40 IIIa	38	−32	34	3.69**	390 2	34	-36	38	3.88*	316 2					
		BA 7/40 IV						34	-28	40	3.37*	316 3					
L	Rostral I. Parietal Lobule I,v	BA 40 II	−58	−44	42	4.38**	87										
R	S. Parietal lobule I/Precuneus I	BA 7 IIIa	26	−64	34	5.77**	508 1	24	−64	36	4.01*	238 1					
L	Precuneus I	BA 7/31 IIIa	−4	−62	32	3.96**	292 1	−8	−66	30	4.03*	577 1					
R	Precuneus	BA 31 IIIa	12	−62	14	3.48**	9	12	-62	18	3.46	61 1					
		BA 31 IV						16	-58	12	3.36	61 2					
R	Rectal gyrus	BA 11 IV											2	28	−14	4.10**	126
L	M. Temporal gyrus	BA 21 IV											−52	−24	−8	4.14**	124
R	M. Temporal gyrus I	BA 21 IIIa	54	−26	−12	4.62**	311 2	54	−26	−12	3.40*	187 3					
		BA 21 II	62	−8	−12	4.25**	311 3										
*Sub-cortical areas*																	
L	Caudatus nucleus (head) I	IIIb	−6	6	0	4.43**	66						−8	4	−2	3.39*	13
R	Lateral Globus Pallidus I	IIIb	16	4	−2	3.51**	160 3						16	6	−2	3.47*	118 2
		IV											20	-6	−6	3.57*	118 1
		IIIb	24	0	−2	4.25**	160 1						24	0	−2	3.36*	118 3
R	Substania Nigra	IV											8	−18	−10	3.45	16
R	Thalamus I	II	8	−8	0	4.19**	160 2										
*Cerebellum*																	
L	Cerebellum (anterior lobe) I	II	−4	−40	−12	4.84**	191 1										

Overview of brain areas with statistically significant cerebral blood flow changes when comparing DID patients to high or low DID simulating controls (CH and CL respectively) for the dissociative identity state main effects.

DID  =  dissociative identity disorder patient group.

CH  =  high fantasy prone DID simulating control group.

CL  =  low fantasy prone DID simulating control group.

I =  *A priori* brain areas based on Reinders et al. (2006) [40].

II =  Brain areas found only in the DID within group analysis.

III =  Brain areas found in the DID within group analysis, in the DID versus CH between group analysis *and* in the DID versus CL between group analysis.

IIIa =  Brain areas found in the DID within group analysis *and* in the DID versus CH between group analysis.

IIIb =  Brain areas found in the DID within group analysis *and* in the DID versus CL between group analysis.

IV =  Brain areas not found in the DID within group analysis *but* appears in the between group analysis DID versus CH *or* DID versus CL.

1 =  first peak voxel in the cluster of the specified size.

2 =  second peak voxel in the cluster of the specified size.

3 =  third peak voxel in the cluster of the specified size.

** =  whole brain multiple comparison correction (*p*<0.05) using false discovery rate statistics [85].

* =  region of interest multiple comparison correction (*p*<0.05) using false discovery rate statistics [85].

u =  Callosomarginal sulcus (SCM) ( =  Cingulate sulcus).

v =  Supramarginal gyrus (Rostral I. Parietal Lobule).

(x, y, z)  =  MNI coordinates in mm.

L/R  =  Left/Right.

kE  =  clustersize in voxels (one voxel is 2×2×2 mm).

NIS  =  neutral identity state.

TIS  =  trauma-related identity state.

BA  =  Brodmann area.

I.  =  inferior; M.  =  middle; S.  =  superior.

### Comparing Simulated and Pathological Identity States

#### Main effects and conjunction analyses

**Figure 2:**
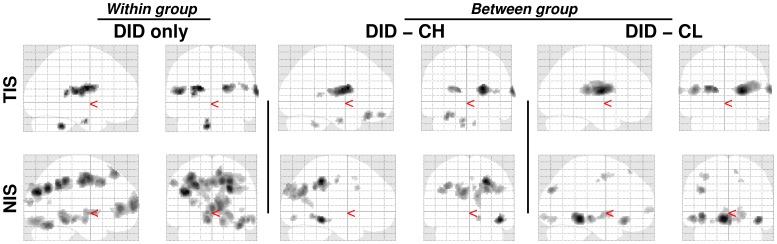
“Glass brain” renderings showing the dissociative identity state main effects, both for the trauma-related identity state (TIS) and for the neutral identity state (NIS), for the dissociative identity disorder (DID) group (left) and the comparison of this group to the high (middle) and low (right) fantasy prone DID simulating controls (CH and CL respectively). See Table 2 for the specific areas.

Results for the within DID group re-analyses and for the two between group comparisons of the dissociative identity states (DIS) main effects, both TIS and NIS, are given in Table 2. Significant differences in rCBF changes between the DID and both the high and low fantasy prone groups were found, i.e., text independent effects. These findings are shown in Figure 2. Commonalities in brain activation between patients and controls were found (data not shown).

#### Trauma-related MS effects within identity state

**Figure 3:**
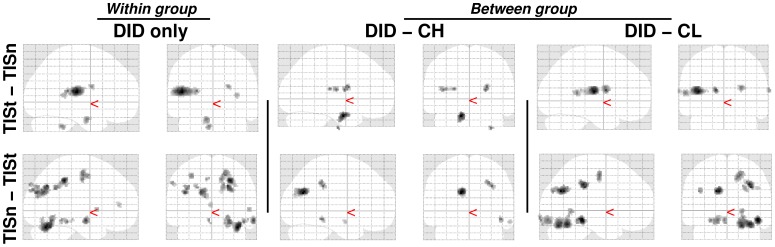
“Glass brain” renderings show differences in the processing of the trauma-related text (indicated with a small ‘t’) and the neutral text (indicated with a small ‘n’) within the trauma-related identity state (TIS). Differences in regional cerebral blood flow patterns for the dissociative identity disorder (DID) group (left) and the comparison of this group to the high (middle) and low (right) fantasy prone DID simulating controls (CH and CL respectively) are depicted. See Table 3 for the specific areas.

**Figure 4:**
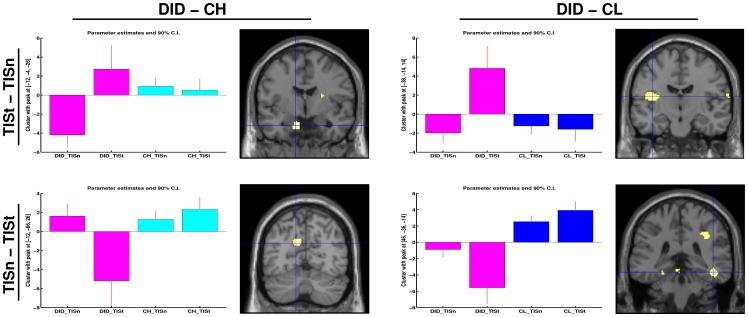
The brain areas indicated with the blue cross (i.e. the peak voxel) are (from top left to bottom right): **the left amygdala, the left insula, the left precuneus, and the right occipitotemporal sulcus.** These areas have the most significant rCBF differences between the dissociative identity disorder patients and high and low fantasy prone DID simulating controls (CH and CL respectively) and is shown both in directionality, i.e. the bar graphs, and location, i.e. shown on a coronal overlay (left in the picture is left in the brain). Results show the differential processing of the trauma-related text versus the neutral text within the TIS, when comparing the DID groups to the high fantasy prone control group (left) and low fantasy prone control group (right).

Trauma-related MS effects within both TIS and NIS are given in Table 3. TIS showed significant regionally specific increases and decreases in cerebral blood flow, when processing the trauma-related MS as compared to the neutral MS, between the DID and both the high and low fantasy prone control groups. These findings are depicted in Figure 3 and 4.

**Table 3:** Memory script effects within dissociative identity state.

				*Within group*					*Between group*									
				DID only					DID - CH					DID - CL				
L/R	Brain Region I = *a priori*		BA	x	y	z	T	kE	x	y	z	T	kE	x	y	z	T	kE
**TISt - TISn**																		
*Cortical areas*																		
L		Insula I	BA 13 II	−46	−20	14	5.32	569 1										
			BA 13 III	−38	−14	14	4.36	569 2	−38	−14	14	3.61*	48 1	−38	−14	14	4.61*	327 1
L		Parietal operculum I	BA 40 IIIb	−48	−30	12	4.02	569 3						−50	−22	12	3.39*	327 2
R		Postcentral gyrus	BA 43 IV											68	−14	18	3.58	19
R		I. Temporal gyrus	BA 20 IV						32	−12	−44	3.57	9					
L		S. Temporal gyrus	BA 42 IV											−68	−28	14	3.51	95 2
			BA 22 IV											−58	−40	12	3.73	95 1
*Sub-cortical areas*																		
L	Amygdala I		IIIa	−10	−6	−24	4.18	63	−12	−4	−26	4.05*	132					
L	Caudate nucleus (dorsal part) I		IV											−12	4	16	4.13*	39
R	Caudate nucleus (dorsal part) I		III	22	0	22	4.05	28	24	−2	14	3.64*	53 1	26	2	20	3.76*	56
R	Caudate nucleus(lateral-dorsal part) I		IV						28	6	14	3.18*	53 2					
L	Caudate nucleus (tail) I		IIIa	−24	−34	16	4.36	20	−22	−24	16	3.60*	13					
R	Caudate nucleus (tail)		II	36	−38	10	3.80	24										
L	Putamen		IV						−24	−18	14	3.30	48 2	−24	−18	14	3.22	327 3
*Cerebellum*																		
L	Cerebellar Tonsil (nodule) I		II	−6	−42	−34	4.07	34										
**TISn - TISt**																		
*Cortical areas*																		
R	Angular gyrus		BA 39 IV											48	−74	32	3.30	11
L	Anterior Cingulate gyrus		BA 32 II	−2	44	8	3.61	14										
L	Posterior Cingulate gyrus I		BA 31 II	−8	−38	46	3.78	11										
R	Cingulate sulcus I,u		BA 6/24 IIIb	20	−10	52	4.02	116 1						20	−10	52	3.76*	101
			BA 6/24 II	14	−12	46	3.90	116 2										
L	Cuneus I		BA 18/19 IIIa	−10	−78	24	3.81	177 2	−10	−78	24	3.39*	210 2					
R	Cuneus		BA 18/19 II	16	−84	28	3.41	64 3										
R	I. Frontal gyrus		BA 44 II	56	10	2	3.69	18										
R	Fusiform gyrus I		BA 19/37 IIIb	26	−56	−14	4.11	345 2						26	−58	−18	3.77*	478 3
			BA 18 IV											26	−96	−20	3.48	38 2
			BA 19/37 IIIb	36	−56	−20	3.80	345 3						34	−58	−20	4.11	478 1
			BA 19/37 II	38	−66	−24	4.65	345 1										
L	Lingual gyrus		BA 18 IV											−14	−88	−14	3.73	139 1
			BA 18 IV											−10	−82	−10	3.23	139 2
R	Lingual gyrus		BA 18 IV											22	−72	−22	4.09	478 2
			BA 18 IV											24	−100	−10	3.65	38 1
L	S. Occipital gyrus I/Angular gyrus		BA 19/39 IIIb	−40	−80	26	4.19	39						−42	−80	28	3.59	29
R	S. Occipital gyrus I		BA 19 II	30	−84	30	3.96	26										
R	S. Occipital sulcus I/Cuneus I		BA 18/19 II	18	−90	38	3.86	64 1										
R	Occipitotemporal sulcus I		BA 20/37 III	48	−34	−16	3.96	34 1	48	−38	−12	3.65	23	46	−36	−14	4.31*	89
L	Parahippocampal gyrus		BA 35/36 IV											−24	−34	−14	3.61	23
L	Intra-Parietal sulcus I		BA 7/40 II	−38	−52	32	3.99	27										
			BA 7/40 II	−22	−48	34	3.81	14										
R	Intra-Parietal sulcus I		BA 7/40 II	28	−38	44	4.54	110 1										
			BA 7/40 III	38	−36	36	3.37	110 2	34	−36	36	3.72*	62	34	−34	38	4.13*	122
L	Rostral I. Parietal Lobule I,v		BA 40 II	−60	−44	40	3.77	19										
R	S. Parietal Lobule/Precuneus I		BA 7 IIIb	24	−64	36	4.32	65						28	−66	32	3.49*	34
L	Precentral sulcus I		BA 4/6 II	−30	−8	52	3.85	43										
L	Precuneus I		BA 7/31 III	−16	−68	28	4.22	177 1	−12	−66	26	4.25*	210 1	−16	−68	28	4.26*	181
			BA 7/31 II	−10	−64	32	3.74	177 3										
R	(Pre-)Cuneus/Parieto-occipital sulcus		BA 7/19 II	18	−78	34	3.79	64 2										
R	M. Temporal gyrus I		BA 21 II	54	−26	−12	3.58	34 2										
			BA 21 IIIa	62	−6	−14	4.24	48	60	−2	−16	3.33	11					
*Cerebellum*																		
L	Cerebellum (anterior lobe)		IIIb	−4	−42	−14	3.79	11						−4	−44	−14	3.62	48 1
			IV											−4	−36	−12	3.43	48 2

Overview of brain areas with statistically significant cerebral blood flow changes when comparing DID patients to high or low DID simulating controls (CH and CL respectively) for the trauma-related memory script effects within dissociative identity state.

DID  =  dissociative identity disorder patient group.

CH  =  high fantasy prone DID simulating control group.

CL  =  low fantasy prone DID simulating control group.

I =  *A priori* brain areas based on Reinders et al. (2006) [40].

II =  Brain areas found only in the DID within group analysis.

III =  Brain areas found in the DID within group analysis, in the DID versus CH between group analysis *and* in the DID versus CL between group analysis.

IIIa =  Brain areas found in the DID within group analysis *and* in the DID versus CH between group analysis.

IIIb =  Brain areas found in the DID within group analysis *and* in the DID versus CL between group analysis.

IV =  Brain areas not found in the DID within group analysis *but* appear in the between group analysis DID versus CH *or* DID versus CL.

1 =  first peak voxel in the cluster of the specified size.

2 =  second peak voxel in the cluster of the specified size.

3 =  third peak voxel in the cluster of the specified size.

** =  whole brain multiple comparison correction (*p*<0.05) using false discovery rate statistics [85].

* =  region of interest multiple comparison correction (*p*<0.05) using false discovery rate statistics [85].

u =  Callosomarginal sulcus (SCM) ( =  Cingulate sulcus).

v =  Supramarginal gyrus (Rostral I. Parietal Lobule).

(x, y, z)  =  MNI coordinates in mm.

L/R  =  Left/Right.

kE  =  clustersize in voxels (one voxel is 2×2×2 mm).

NISn  =  neutral identity state exposed to the neutral memory script.

NISt  =  neutral identity state exposed to the trauma-related memory script.

TISn  =  trauma-related identity state exposed to the neutral memory script.

TISt  =  trauma-related identity state exposed to the trauma-related memory script.

BA  =  Brodmann area.

I.  =  inferior; M.  =  middle; S.  =  superior.

#### Trauma-related MS effects between identity state

**Figure 5:**
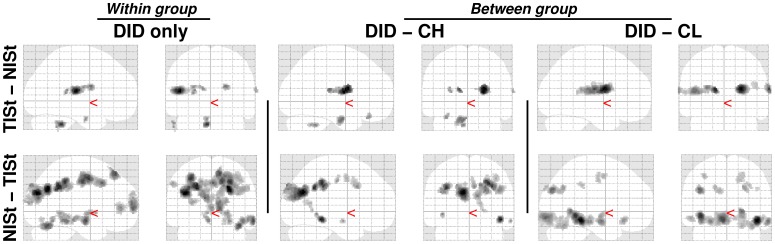
“Glass brain” renderings show differences in the processing of the trauma-related text (indicated with a small ‘t’) between the trauma-related identity state (TIS) and the neutral identity state (NIS). Differences in regional cerebral bloodflow patterns for the dissociative identity disorder (DID) group (left) and the comparison of this group to the high (middle) and low (right) fantasy prone DID simulating controls (CH and CL respectively) are depicted. See Table 4 for the specific areas.

**Figure 6:**
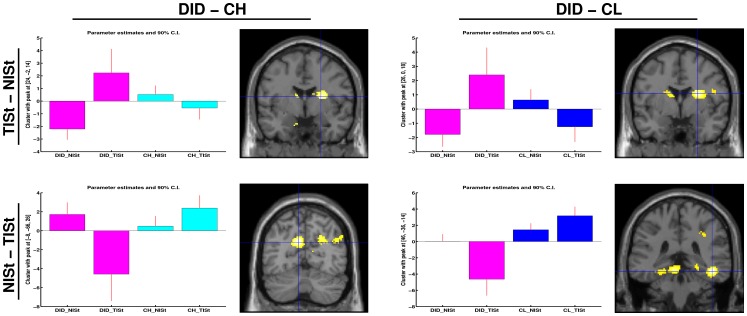
The brain areas indicated with the blue cross (i.e. the peak voxel) are (from top left to bottom right): the right caudate nucleus (dorsal part) (2x), the left precuneus, and the right occipitotemporal sulcus. These areas involve the most significant rCBF difference between the dissociative identity disorder patients and high and low fantasy prone DID simulating controls (CH and CL respectively) and is shown in both directionality, i.e. the bar graphs, and location, i.e. shown on a coronal overlay (left in the picture is left in the brain). Results show the differential processing of the trauma-related text between the TIS and the NIS, when comparing the DID groups to the high fantasy prone control group (left) and low fantasy prone control group (right).

Trauma-related MS effects between DIS are given in Table 4. Different rCBF patterns were found for NIS and TIS, when processing the trauma-related MS, between the DID and both the high and low fantasy prone control groups. These differential rCBF patterns are shown in Figure 5 and 6. The results indicate that, for some areas (e.g. the parahippocampal gyrus in the comparison NISt-TISt or the caudate nucleus in the comparison TISt-NISt), the difference in blood flow between patients and controls is larger than the difference between the DID identity states.

**Table 4:** Memory script effects between dissociative identity states.

				*Within group*										*Between group*																				
				DID only										DID - CH										DID - CL										
L/R	Brain region I = *a priori*	BA		x		y		z		T		kE		x		y		z		T		kE		x		y		z		T		kE		
**TISt-NISt**																																		
*Cortical areas*																																		
L	Insula I	BA 13 IIIb		−46		−20		14		5.32		367 1												−42		−20		16		4.10**		568 2		
		BA 13 II		−34		−16		16		3.65		367 2																						
R	Insula	BA 13 IV																						42		−6		26		3.26		37 2		
		BA 13 IV																						46		−12		26		3.41		16		
L	Orbitofrontal cortex	BA 11 IV												−32		36		−22		3.58		21												
R	Parietal operculum	BA 40/43 IV																						48		−2		22		3.40		37 1		
R	Postcentral gyrus I	BA 43 III		68		−14		14		3.83		23		68		−14		14		3.44		12		68		−14		18		4.45**		106		
R	Precentral gyrus	BA 6 IV																						66		8		20		3.87		41		
L	S. Temporal gyrus	BA 22/42 IV																						−68		−28		14		3.81		92 1		
		BA 22 IV																						−58		−40		14		3.45		92 2		
*Sub-cortical areas*																																		
L	Amygdala I	IV												−16		−12		−32		3.34*		129 2												
		IIIa		−6		−6		−26		4.06		24		−6		−6		−26		3.75*		129 1												
R	Caudate nucleus (caudal part) I	IIIa		20		−26		20		4.01		10		20		−24		18		3.75*		352 3												
L	Caudate nucleus (dorsal part) I	III		−26		−10		20		4.07		78 1		−26		−10		20		3.49*		29 2		−26		−4		20		4.02**		568 3		
		III		−12		2		18		4.04		78 2		−12		2		18		3.90*		34		−12		4		16		4.71**		568 1		
R	Caudate nucleus (dorsal part) I	IV												24		−2		14		4.38*		352 1		26		0		18		5.09**		490 1		
		IIIa		22		2		22		4.29		61		24		4		20		4.37*		352 2												
L	Caudate nucleus (tail) I	II		−24		−34		16		4.12		19																						
L	Putamen	IV												−24		−18		14		3.55		29 1												
R	Putamen	IV																						26		−14		16		3.66		490 2		
*Cerebellum*																																		
L	Cerebellar Tonsil (nodule) I	IV												−8		−56		−34		3.25*		47 2												
		IIIa		−6		−44		−34		4.81		84		−6		−46		−34		3.63		47 1												
L	Cerebellum (lateral part) I	II		−56		−48		−32		4.03		24 1																						
		II		−54		−48		−42		3.41		24 2																						
**NISt-TISt**																																		
*Cortical areas*																																		
R	Angular gyrus	BA 39 III		48		−64		32		4.03**		597 2		44		−62		30		3.80		135 1		46		−62		34		3.34		11		
		BA 39 IV												50		−66		34		3.44		135 2												
L	Anterior Cingulate gyrus	BA 32 II		−2		38		10		3.62**		142 2																						
		BA 32 II		−4		54		10		3.34**		142 3																						
X	Anterior Cingulate gyrus I	BA 32 II		0		46		6		4.21**		142 1																						
L	Cingulate gyrus I	BA 24 II		−10		−12		38		3.96**		40																						
R	Cingulate gyrus I	BA 32 IIIa		2		18		42		4.64**		376 1		8		14		36		3.74*		89 1												
		BA 32 IIIa		6		8		48		3.80**		376 3		4		8		50		3.28*		89 2												
L	Cingulate sulcus/Cingulate gyrus I	BA 24/32 II		−16		12		44		4.10**		376 2																						
R	Cingulate sulcus I,u	BA 6/24 IIIb		16		−12		44		4.91**		614 1												20		−10		46		3.53*		72 1		
		BA 6/24 II		20		−10		52		4.51**		614 3																						
L	Posterior Cingulate gyrus	BA 31 IV												−6		−42		40		3.37		14												
L	Cuneus I	BA 18/19 III		−10		−78		20		4.57**		620 1		−10		−78		20		4.08*		921 2		−12		−72		30		3.64*		92 1		
		BA 18/19 III		−14		−72		30		4.17**		620 2		−14		−76		32		3.92*		921 3		−16		−90		36		3.56*		20		
R	Cuneus I	BA 18/19 IIIa		16		−84		28		3.83**		212 3		12		−80		22		3.86*		252 1												
		BA 19 IIIa		18		−80		36		3.99**		212 2		18		−90		38		3.80*		252 2												
		BA 19 IV												18		−84		30		3.52*		252 3												
X	Cuneus	BA 18 II		0		−90		40		3.61**		19																						
L	I. Frontal gyrus	BA 47 IV																						−42		28		−12		3.42		19		
R	S. Frontal gyrus I	BA 10 II		22		66		16		4.03**		407 2																						
		BA 8 II		12		38		56		4.83**		177 1																						
		BA 6 II		14		26		66		3.40**		177 2																						
R	S./Medial Frontal gyrus I	BA 10 II		12		64		4		4.59**		407 1																						
R		BA 10 II		10		64		20		4.02**		407 3																						
L	S. Frontal sulcus I	BA 6 IIIb		−32		−2		48		5.02**		318												−34		−2		52		3.64		36		
R	S. Frontal sulcus I	BA 4/6 IV												26		−14		52		3.32*		33 2		24		−10		56		3.44*		72 2		
		BA 4/6 IIIa		28		−14		46		4.56**		614 2		28		−16		44		3.61*		33 1												
L	Fusiform gyrus	BA 19 IV																						−28		−68		−10		3.47		28		
R	Fusiform gyrus I	BA 18 IV																						26		−96		−20		3.34*		34 1		
		BA 19/37 II		26		−56		−14		4.10**		149 1																						
		BA 19 II		40		−78		−22		3.38**		185 2																						
		BA 19/37 IIIb		42		−64		−20		4.79**		185 1												38		−62		−20		3.79**		482 2		
L	Lingual gyrus	BA 18 IV																						−10		−84		−14		3.88**		616 3		
		BA 18 IV																						−6		−82		−6		3.91**		616 2		
		BA 18 IV																						−4		−90		−10		4.33**		616 1		
R	Lingual gyrus	BA 18 IIIb		20		−72		−14		3.68**		26												18		−98		−14		3.20		34 2		
L	M. Occipital gyrus I	BA 18 II		−30		−92		12		3.99**		364 2																						
		BA 18 II		−24		−98		16		3.73**		364 3																						
L	S. Occipital gyrus I/Angular gyrus	BA 19/39 III		−38		−80		28		5.61**		364 1		−38		−82		30		3.72*		82		−42		−78		32		4.27**		128		
R	S. Occipital gyrus	BA 19 II		30		−84		30		4.02**		597 3																						
R	S. Occipital sulcus I/Cuneus	BA 18/19 II		18		−90		38		4.35**		212 1																						
R	Occipitotemporal sulcus I	BA 20/37 III		46		−34		−14		4.39**		327 2		48		−40		−12		4.53*		92		46		−36		−14		5.24**		294 1		
L	Parahippocampal gyrus I	BA 35 IV												−40		−46		−4		3.75		18		−40		−46		−6		4.73**		780 1		
		BA 35/36 IIIb		−12		−42		−6		3.59**		70 2												−24		−34		−14		4.02**		780 3		
R	Parahippocampal gyrus I	BA 36 IV												20		−52		2		3.46*		52 1		22		−52		0		4.26**		482 1		
		BA 35/36 IIIb		20		−46		−2		4.03**		149 2												22		−42		−6		3.77**		482 3		
		BA 35/36 II		24		−34		−6		3.98**		149 3																						
L	Intra-Parietal sulcus I	BA 7/40 IIIa		−38		−52		32		4.96**		426 2		−34		−50		34		4.36*		117												
		BA 7/40 II		−22		−48		34		5.25**		426 1																						
		BA 7/40 II		−22		−36		38		4.53**		426 3																						
R	Intra-Parietal sulcus I	BA 7/40 II		28		−38		44		5.07**		332 1																						
		BA 7/40 III		34		−36		38		4.15**		332 2		30		−38		40		4.36*		249		34		−34		38		3.52		30		
L	Rostral I. Parietal lobule I,v	BA 40 II		−58		−44		42		4.00**		52																						
R	S. Parietal lobule/Precuneus I	BA 7 III		26		−64		32		5.61**		597 1		24		−64		30		3.95*		108		24		−64		36		3.80**		48		
L	Precuneus I	BA 7/31 III		−6		−62		30		3.85**		620 3		−8		−66		26		4.72		921 1		−10		−64		32		3.53*		92 2		
R	Precuneus	BA 31 IV												14		−64		16		3.25		52 3												
		BA 31 IV												16		−54		12		3.42		52 2												
X	Rectal gyrus	BA 11 IV																						0		28		−12		3.82**		85		
L	M. Temporal gyrus	BA 21 IV																						−54		−24		−10		3.71**		63		
R	M. Temporal gyrus I	BA 21 III		62		−6		−14		4.80**		327 1		62		−6		−14		3.64		15		62		−6		−14		4.16**		120		
		BA 21 IIIb		54		−26		−12		4.24**		327 3												54		−24		−10		3.66**		294 2		
*Sub-cortical areas*																																		
L	Caudatus nucleus (head) I	II		−6		8		−2		3.94**		15																						
R	Lateral Globus Pallidus I	IV																						24		−8		−8		3.77**		85 1		
		IIIb		24		0		−2		4.13**		70 1												24		0		−2		3.34*		85 2		
R	Medial Globus Pallidus	II		14		−6		−2		3.44**		70 2																						
*Cerebellum*																																		
L	Cerebellum (anterior lobe)	IIIb		−6		−42		−12		4.23**		70 1												−4		−42		−12		4.53**		780 2		

Overview of brain areas with statistically significant cerebral blood flow changes when comparing DID patients to high or low DID simulating controls (CH and CL respectively) for the trauma-related memory script effects between dissociative identity state.

DID  =  dissociative identity disorder patient group.

CH  =  high fantasy prone DID simulating control group.

CL  =  low fantasy prone DID simulating control group.

I =  *A priori* brain areas based on Reinders et al. (2006) [40].

II =  Brain areas found only in the DID within group analysis.

III =  Brain areas found in the DID within group analysis, in the DID versus CH between group analysis *and* in the DID versus CL between group analysis.

IIIa =  Brain areas found in the DID within group analysis *and* in the DID versus CH between group analysis.

IIIb =  Brain areas found in the DID within group analysis *and* in the DID versus CL between group analysis.

IV =  Brain areas not found in the DID within group analysis *but* appear in the between group analysis DID versus CH *or* DID versus CL.

1 =  first peak voxel in the cluster of the specified size.

2 =  second peak voxel in the cluster of the specified size.

3 =  third peak voxel in the cluster of the specified size.

** =  whole brain multiple comparison correction (*p*<0.05) using false discovery rate statistics [85].

* =  region of interest multiple comparison correction (*p*<0.05) using false discovery rate statistics [85].

u =  Callosomarginal sulcus (SCM) ( =  Cingulate sulcus).

v =  Supramarginal gyrus (Rostral I. Parietal Lobule).

(x, y, z)  =  MNI coordinates in mm.

L/R  =  Left/Right.

kE  =  clustersize in voxels (one voxel is 2×2×2 mm).

NISn  =  neutral identity state exposed to the neutral memory script.

NISt  =  neutral identity state exposed to the trauma-related memory script.

TISn  =  trauma-related identity state exposed to the neutral memory script.

TISt  =  trauma-related identity state exposed to the trauma-related memory script.

BA  =  Brodmann area.

I.  =  inferior; M.  =  middle; S.  =  superior.

## Discussion

The present study was performed to examine whether earlier reported results [39], [40] for DID hold after correcting for potential iatrogenic and other sociogenic effects. To this end, we tested whether these findings can be simulated by motivated role-enactment and/or is facilitated by a high level of fantasy proneness [18] by re-investigating the patient population from Reinders et al. [39], [40]. Results of a sub-study (see Methods and Supporting Information S1) show that DID patients have a fantasy proneness score of 9.83 (SD 5.25), which approximates the normal population, indicating that fantasy proneness might not play a major role in DID. This finding is consistent with the current psychobiological results. Neither high nor low fantasy prone healthy controls, instructed and motivated to simulate two different types of dissociative identity states in DID (i.e., NIS and TIS), mimicked previously observed psychophysiological and neural reactions that are associated with these identity states in DID [39], [40], which is supportive of our first *a priori* hypothesis.

From results shown in Figures 2, 3, and 5 a general feel of the effects can be obtained. Figures 2 and 5 and the top row of Figure 3 show that in the high fantasy prone control group more of the original DID rCBF patterns are apparent, while the low fantasy prone control group show less similarities with the original DID rCBF patterns, for example the disappearance of the left amygdala activation. Less similarities between patients only and patients versus controls means more overlap in rCBF patterns. In other words the less differences in the rCBF patterns between patients only and patients versus controls, the better the controls simulate DID. Thus, relatively speaking, low fantasy prone controls simulated the performance of DID patients better than high fantasy prone controls. This result is the opposite from the direction indicated by holders of the sociocognitive and fantasy based model of DID [17], [19]–[21], [46]. As patients and controls were scanned in a highly similar experimental setting and because controls were highly motivated to simulate DID, commonalities in brain activation between patients and controls were expected. Despite the overlap in brain activation between patients and controls important previously found psychophysiological and neurobiological differences between NIS and TIS in DID patients were upheld when controlling for fantasy proneness, suggestion, and instructed and motivated role-playing, which is supportive of our first *a priori* hypothesis.

The activated areas seem to be subdivided in two distinct neural networks, where the NIS activates areas in the cerebral cortex, while the TIS mainly activates subcortical areas (e.g., see Table 2 and Figure 2). The Tables show a detailed listing of all the brain areas involved. The brain areas marked with a ^II^ in the Tables are brain areas non-specific to DID as they disappear after comparing to a control group, i.e. these areas share commonalities between patients and controls. The brain areas marked with a ^III^ and *^IV^* in the Tables are brain areas specific to DID. The areas in the latter group are areas that were not reported earlier as they were “subtracted out” in the within group comparisons.

Our findings support the cortico-limbic inhibition model of trauma-related dissociative disorders [41], [47]. Results of both the NISt-TISt comparison and the main effect of NIS show significant overlap with the activated network of brain regions during emotional memory suppression of unwanted memories in mentally healthy individuals [48], for example in frontal areas (BA 4/6/8/10/47), cingulate cortex (BA 32), and intraparietal sulcus (BA 7/40). Anderson et al. [48] did not find all of these brain areas. There is significant overlap between our study and their study, but the brain areas involved in the modulation of access to trauma-related memory in our patient population are of larger number. This might be an indication that, when functioning as NIS, in DID patients different cortical processes are involved that modulate conscious and subconscious perception of trauma-related information. These areas, e.g. (pre-)cuneus (BA 7/39, 18/19), fusiform gyrus (BA 18/19/37), lingual gyrus (BA 18), occipital gyrus (BA 18/19/37), and the parahippocampal gyrus (BA 35/36), are located in the posterior association areas (PAA) and have been indicated to be involved in multimodal [49], [50] somato-sensory integration [51], [52] of information, especially in relation to attention and perceptual awareness [49]. Hyperactivation of cortical multimodal association areas for NIS in DID when listening to personal trauma scripts constituted our third *a priori* hypothesis. We thus propose that for emotional memory suppression, or NIS’ mental avoidance [41], of unwanted memories in DID the PAA fulfils a pivotal role.

There are notable similarities in the patterns of brain activation for DID patients, as revealed in the main effect TIS and the TISt-NISt comparison, and mentally healthy individuals unsuppressed memory retrieval [48]. Both groups had increased activation of the insula (BA 13) and parietal operculum (BA 40/43). We did not find the hippocampus to play a role in memory retrieval in DID patients, despite the fact that this area has been indicated in memory processing in mentally healthy individuals [48]. Instead we found that the caudate nucleus was activated when DID patients listened to the trauma-memory scripts as TIS. Acute stress can be associated with a shift from hippocampal involvement to caudate nucleus involvement [53], [54]. Thus, acute stress is linked with caudate nucleus-dependent stimulus-response type reactivity at the expense of hippocampal dependent spatial learning and memory [53]. According to the theory of structural dissociation [33], [41] listening to a description of a personal traumatic memory in an experimental setting constitutes a consciously experienced acute stressor for TIS, because as this dissociative identity state DID patients do not manage to mentally avoid the relevant memory. When DID patients as TIS are confronted with reminders of traumatic memories, they may initiate a caudate mediated reflex-like flight-fright-freeze response [55], [56] which reaction is also supported by an accompanying amygdala activation [44], [57]. Another, but compatible, explanation for increased caudate and amygdala activation in DID patients as compared to controls is a heightened memory sensitivity for negative valanced information [58]. These findings for TIS are supportive of our second *a priori* hypothesis.

To date, experimental research of inter-identity amnesia in DID has produced mixed results. One study [59] demonstrated evidence for inter-identity amnesia, which is in line with the current findings. Other studies [60]–[65] found inter-identity transfer of newly learned non-autobiographical stimuli, even though the “amnestic” identity reported subjective amnesia for these stimuli. Several principles might explain the inconsistent findings: (i) Inter-identity amnesia may only exist for stimuli that have personal relevance for the “amnestic” identity. In the cited studies [59]–[65], it was not assessed if or to what degree the applied stimuli had autobiographical meaning for the tested “amnestic” and “mnestic” dissociative identities. Our study included traumatic memories that were subjectively autobiographical for TIS but not for NIS, and found that NIS and TIS had different subjective, psychophysiological, and neural reactions to a description of the involved traumatic memories. We also found that as a NIS, DID patients did not relate these traumatic memories to themselves [39]. These results indicate the importance of using autobiographical information when investigating inter-identity amnesia in DID. (ii) Inter-identity amnesia may predominantly exist between different types of dissociative identities, particularly between neural and trauma-related identity states.This has been clinically observed, theoretically proposed [33], [41] and is in line with our results. Unfortunately, in most studies [59]–[66] it was not assessed what types of dissociative identities participated, e.g. NIS or TIS. Therefore, we strongly recommend that in future research in DID the types of dissociative identities are verified and reported and that test material is used that is subjectively autobiographical for one dissociative identity, but not for another.

The sociocognitive view of DID entails the idea that this disorder can be easily and readily created in motivated suggestible individuals and that few suggestions would suffice to generate the symptoms of DID [15] (see Supporting Information S2). Still, one might argue that the current brief practice of DID simulation is insufficient to simulate the psychobiological profiles of NIS and TIS. Even if years of practice could generate these profiles, our findings suggest that fantasy proneness is not the driving factor because low fantasy prone controls simulated the performance of DID patients better than high fantasy prone controls. This result is the opposite from the direction indicated by holders of the sociocognitive and fantasy based view. Therefore we feel that our study provides an important contribution to the etiology discussion.

For the first time, it is shown using brain imaging that neither high nor low fantasy prone healthy women, who enacted two different types of dissociative identity states, were able to substantially simulate these identity states in psychobiological terms. These results do not support the idea of a sociogenic origin for DID.

## Methods

### Participants

#### Controls

Mentally healthy females were recruited by local newspaper advertisements. Respondents were sent a letter in which the study was explained and in which they were invited to complete three questionnaires: (i) the Traumatic Experiences Checklist (TEC) [67], a self-report questionnaire assessing potentially traumatizing events such as physical abuse and emotional neglect, (ii) the Somatoform Dissociation Questionnaire (SDQ-20 [68]–[70], a self-report questionnaire evaluating the severity of somatoform dissociative symptoms, e.g., analgesia, anesthesia, motor inhibitions), and (iii) the Creative Experiences Questionnaire (CEQ) [18] which measures fantasy proneness. Exclusion criteria were the presence of medical, neurological or psychiatric problems in the past or the present, the use of psychotropic medication 15 days prior to examination, participation in a positron emission tomography (PET) or other study that involved administration of radiation in the year prior to this study, and pregnancy. A total of 18 healthy controls participated in the study, which was approved by the Medical Ethical Committee of the University Medical Center Groningen.

After inclusion, written and oral information on dissociative identity states (i.e. NIS and TIS) in DID and instructions on how to simulate these dissociative identity states was given to the controls. It was checked whether the controls understood this information. A template for training themselves in switching between the simulated identity states was provided. Controls were then questioned about how they constructed the two identity states, whether they encountered difficulties and if so, they were given support to improve their roles as NIS and TIS. To help the controls simulate NIS and TIS, they were asked to recall two experiences they had had earlier in their life, an emotionally neutral experience and an emotionally painful experience. Controls were asked to provide their most painful memory to serve as an analogue for the patients’ personal trauma memories, as well as a neutral personal episodic memory. Controls were subsequently instructed how to write the autobiographical analogue neutral and “trauma” memory scripts. For the experiment they had to train themselves in being in a neutral state, the NIS who is unresponsive or under-responsive to the painful experience, and in being in a state in which they re-experience the painful memory, the TIS. The consecutive and final check on the capability to simulate the two different dissociative identity states consisted in checking whether their description of their neutral and painful experiences (that was to be casted in an audiotape recording) met the instructions on how to enact a DID patient.

In the two or more weeks preceding the PET scans, candidate control subjects practiced simulating NIS and TIS, as well as alternating between NIS and TIS using detailed role instructions. One of the investigators (H.V.) contacted the candidates per telephone during this preparatory phase to ensure that they followed the instructions and to offer further suggestions for optimizing their role performance. One candidate felt unable to simulate the roles satisfactorily, and was therefore excluded. Prior to the actual PET scanning, H.V. checked if the candidates experienced and judged that they were able to simulate the roles of NIS and TIS. During the actual scanning, he checked if they engaged in the requested simulations, and immediately after the role performances, he checked if the controls generally felt they had simulated the roles of NIS and TIS effectively. All controls passed these various checks. In addition, immediately after each text condition, H.V. administered a detailed questionnaire that inquired after the controls’ subjective sensorimotor and emotional experiences during their role performance. This questionnaire was identical to the one in the patient study [39], [40], which was administered by the patients’ therapist, and debriefed six subjective emotional experiences (fear, sorrow, sadness, anger, shame and disgust) and ten sensorimotor experiences (visual, kinesthetic, auditory, olfactory + gustatory reactions, pain, physical numbness, body stiffening, paralysis and restlessness) were debriefed. In addition, the presence of the identity state under investigation and the interference among identity states were also debriefed. Using this questionnaire, H.V. or the patients’ therapist could structurally evaluate if the intended NIS or TIS had been present during the experimental condition. Statistical analyses of the simulation performance in terms of their subjective experiences, i.e. the subjective sensorimotor perception and emotional response, during the scanning by the two control groups are provided in Supporting Information S2.

As we did not have CEQ values for the patients (see also Supporting Information S1) we could not control for fantasy proneness by including a covariate. Therefore, the controls were divided into two groups based on their CEQ scores resulting in a high fantasy prone group (n  = 10, age 38.2 (SD 10.9), TEC 0.7 (SD 1.3), SDQ-20 22 (SD 2.4)) with CEQ 13.7 (SD 3.2) and a low fantasy prone group (n  = 8, age 42.5 (SD 10.1), TEC 0.4 (SD 0.5), SDQ-20 20.9 (SD 1.5)), with CEQ 3.9 (SD 1.6). A CEQ cut-off for high fantasy proneness of 10 was used, which the developers of the CEQ recommended for the current sample [71].

### Patients

A detailed description of the DID patients can be found elsewhere [39], [40]. In short: Eleven patients (all female, age 41.0, SD 6.1) participated: (i) whose treatment had progressed to Phase II [72], which involves therapeutic exposure to trauma-related memories, (ii) who met criteria for DID, as operationalized in the Structured Clinical Interview for DSM-IV Dissociative Disorders (SCID-D [73]), and (iii) had at least one TIS and one NIS that they could activate on demand [33] and (iv) the involved TIS had displayed signs of sympathetic nervous system dominance under perceived threat in clinical situations.

To establish the CEQ values in DID patients an independent and representative sample of DID patients (n = 42) completed the CEQ. Details regarding this substudy can be found in the Supporting Information S1.

### Stimulus Scripts

During scanning, patients and controls listened to descriptions of the neutral episodic memories and memories of traumatizing or most painful events that only TIS experienced as a personal memory [74]. These memories were cast, prior to the PET session, by the therapist or one of the principal investigators (H.V.) in terms of stimulus descriptions, and were subsequently audio-taped in a neutral tone of voice as 120 second scripts for playback during the PET investigation.

### PET Procedure

The PET (Siemens/CTI ECAT HR+) procedure for the controls was close to identical to the patients [39], [40]. In contrast to patients the controls did not habituate to the PET environment prior to the investigation as anxiety levels were expected to be low. Approximately two hours prior to the PET investigation the continuous ECG registration was started, obtaining the five frequency and time domain variables [75], [76]. No urine samples were obtained for the control groups, both medication and drugs use were verbally debriefed according to standard control research practice.

For the controls one extra set of the four conditions was added to increase statistical power. The scanning sequence was therefore NISn, NISt, TISn, TISt, TISn, TISt, NISn, NISt, TISn, TISt, NISn and NISt. The last minor character (n or t) denotes the content of the memory script (MS: neutral or trauma-related). For patient comfort considerations, i.e. minimizing the number of identity state switches, a fixed condition order was used, which was also used for the controls to minimize methodological differences.

Immediately following the end of each script, blood pressure (systolic and diastolic) and discrete heart rate frequency were measured and the six subjective emotional and ten sensorimotor experiences were debriefed. Finally, the presence of the identity state under investigation and the interference among identity states were also debriefed.

### Image Acquisition and Data Processing

Data acquisition, reconstruction, attenuation correction, spatial transformation, spatial smoothing (isotropic Gaussian kernel of 12 mm) and global normalization were performed as usual [39], [40], [77]. SPM5 (www.fil.ion.ucl.ac.uk/spm) was used for spatial transformation to the MNI template (using heavy regularization) [78], [79] and statistical analysis [80] of both patient and control data.

### Data Analysis: Autonomic and Subjective Reactions

Statistical analysis, missing value analysis and principal components (PC) analysis were performed with SPSS-PC 15.0 (2006) in an identical manner as was done for the patient data [39], [40]. Results with *p*<0.05 are reported as significant. Within SPSS two two-by-two-by-two factorial design were defined with the first factor Group, consisting of the levels DID and the high fantasy prone controls or the low fantasy prone controls, a second factor identity state, consisting of the levels NIS and TIS, and the third factor was MS, consisting of the levels neutral and trauma-related. For one high fantasy prone and one low fantasy prone subject heart rate variability (HRV) data could not be obtained. In addition, the data, including the PET data, from two NISt conditions was removed as the control subjects reported not to be able to maintain as a NIS. One TISn condition was removed from the low fantasy prone data as the subject reported not to be able to maintain a TIS. Bonferoni correction to correct for multiple testing was applied.

### Data Analysis: PET-data

The patient PET data included in the current study is identical to the data as included and described in our previous publications [39], [40]. This study assessed various effects, e.g., main effects and simple subtraction analyses (within and between identity state) within the DID group using SPM99. This data was re-analyzed in SPM5 and is referred to as the “within DID only” analyses.

From the 10 high fantasy prone healthy controls the PET data of one subject was lost due to storage failure at the PET center. The data of the three groups was statistically analyzed in SPM5 in a three-by-two-by-two factorial design [81]–[84]. The general linear model (GLM) consisted of the three factor main effects, the four conditions and a group by condition interaction.

In addition, the subjective reactions and the autonomic reactions were included as group specific covariates of interest after PC analysis [39], [40]. The variance in the subjective ratings could be described with the first two, six, and five PC for the DID, high and low fantasy prone groups respectively, explaining 64%, 68%, and 72% of the variance. The variance in the autonomic reactions could be described with the first three PC for each of the DID, high and low fantasy prone groups, explaining 85%, 82%, and 87% of the variance respectively. Finally, the global cerebral blood flow (CBF) was included as a nuisance covariate (AnCova by subject).

### Hypothesis Testing

Previously reported significant findings were tested using a between group subtraction of the within group results (e.g. DID(TISt-NISt)-Control(TISt-NISt)). Commonalities in brain activation between patients and controls were tested using global null conjunction analyses [83].

### Statistical Inference and Reporting

Our *a priori* hypothesis was that earlier findings would still hold after the correction for non-trauma-related factors. Both whole brain and *a priori* region of interest (ROI) multiple comparisons correction were performed on the basis of false discovery rate statistics [85]. Statistical parametric maps were thresholded using an uncorrected threshold of *p*<0.001 [40], [86] and explored for *a priori* hypothesized brain areas. If an *a priori* hypothesized brain area did not survive whole brain multiple comparison correction, multiple comparisons correction was performed within the *a priori* region of interest (ROI). For subcortical located ROI and ROI in the cerebellum a sphere with a volume of 3054 mm^3^ [87] was used. For larger cortical *a priori* hypothesised ROI a sphere with a volume of 6108 mm^3^ was used. Note: in line with previously used statistical thresholds [40] voxels surviving significant levels only uncorrected for multiple testing for the whole brain, i.e. *p*<0.001 [40], [86] were reported as well, but for comparison purposes only. Only clusters larger than eight voxels are reported taking into account the spatial resolution of the PET camera. In contrast to the earlier publication [40], this time all peak voxels are reported for a more accurate comparison between groups.

The coordinates were converted from MNI space to Talairach space [88] to be defined in Brodmann areas (BA) using both the Talairach atlas [79] and Deamon [89], [90]. Activations in sulci was defined using Brain Tutor [91]. The location was anatomically compared to and described using a second brain atlas [92].

## Supporting Information

Supporting Information S1 Fantasy proneness in dissociative identity disorder.
(DOC)

Supporting Information S2 How well are the dissociative identity disorder simulating healthy controls doing?
(DOC)
