# Correction: Fact or Factitious? A Psychobiological Study of Authentic and Simulated Dissociative Identity States

**DOI:** 10.1371/annotation/4f2000ce-ff9e-48e8-8de0-893b67efa3a4

**Published:** 2012-07-12

**Authors:** A. A. T. Simone Reinders, Antoon T. M. Willemsen, Herry P. J. Vos, Johan A. den Boer, Ellert R. S. Nijenhuis

The name of author A.A.T. Simone Reinders contained an error in the citation. The correct citation should read: Reinders AATS, Willemsen ATM, Vos HPJ, den Boer JA, Nijenhuis ERS (2012) Fact or Factitious? A Psychobiological Study of Authentic and Simulated Dissociative Identity States. PLoS ONE 7(6): e39279. doi:10.1371/journal.pone.0039279 

