# Supplementary material for: Fact or Factitious? A Psychobiological Study of Authentic and Simulated Dissociative Identity States
Source: PLoS One. 2012 Jun 29;7(6):e39279. doi: 10.1371/journal.pone.0039279 (PMC3387157; doi:10.1371/journal.pone.0039279)
Supplement: Supporting Information S1 — Fantasy proneness in dissociative identity disorder. (DOC) [file pone.0039279.s001.doc]

**Supporting Information S1**

**Fantasy proneness in dissociative identity disorder**

Ellert R. S. Nijenhuis^1^, PhD & A. A. T. Simone Reinders^2,3^, PhD

*^1^ Top Referent Trauma Center Mental Health Care Drenthe, Assen, The Netherlands*

*^2^ King's College London, Institute of Psychiatry, Department of Psychosis Studies, London, United Kingdom,*

*^3^ Department of Neuroscience, University Medical Center Groningen, and BCN Neuroimaging Center, University of Groningen, Groningen, The Netherlands*

**Introduction**

It has been claimed that dissociative identity disorder (DID) is caused by or at least strongly related to high fantasy proneness[1–3]. A related idea is that the memories of childhood traumatization that DID patients typically report are fantasy-based pseudo-memories[4]. However, these hypotheses have not been tested in patients with DID. In this study, we examined fantasy proneness in a sample of carefully diagnosed cases of DID, and explored the contents of these patients’ fantasies, as well as their subjective motives to engage in fantasies.

**Method**

Fantasy proneness was measured with the Creative Experiences Questionnaire (CEQ[5]). The test-retest stability, internal consistency, and concurrent validity are adequate. The range of possible scores is 0-25. To evaluate the severity of fantasy proneness in DID, the CEQ was administered to 42 women with DID (age M= 42.8, SD 7.0, range 29-62). The presence of DID was assessed by clinical experts using the SCID-D[6]. The sample consisted of women who were in an assessment phase upon their referral to an outpatient trauma clinic, who were in outpatient treatment, who participated in research projects, or who came for a second opinion regarding their diagnosis.

**Results**

*Creative Experiences Score*

The CEQ score for these 42 patients was M=9.83, SD 5.25.

*Fantasies: content and subjective function*

The typical fantasies concerned living in a better, less neglectful and less abusive personal world. All patients used their fantasies to cope with the actual and recalled traumatization. Some reported imagining that the neglect and abuse were not real, or that they were not touched by it. None reported fantasizing about traumatic incidents. However, some had strong, although mistaken ideas that particular (alleged) perpetrators were still alive, that (alleged) perpetrators might be entering the therapy room anytime, that these individuals always know where they (i.e., the patient) are, etcetera.

Typical examples of responses from DID patients to some of the CEQ-items include the following:

* CEQ-item 1: As a child I thought that dolls, teddy bears and stuffed animals, with which I played, were living creatures: “As a child, I was strongly neglected. I thought my dolls needed me.”

* CEQ-item 2: As a child I strongly believed in the existence of dwarfs, elves and other fairy tale figures: “I had to believe this to survive. I hoped my inner world would become visible, so that my inner friends would protect me from the perpetrators.”

* CEQ-item 3: As a child I had my own make believe friend or animal: “To protect myself, I immersed myself in books, where live was better. I knew as a child that my inner world was not objectively real, but when I stepped into it, it was real for me. It had to be.”

* CEQ-item 5: As a child I sometimes had the feeling that I was someone else (e.g., a princess, an orphan, etc.): I believed a was an orphan rather than a member of the family of origin; I did not want to belong to them.” One patient, as one of her TIS, believed that her real mother was looking for her but could not find her. She wanted the therapist to find her real mother and she refused to believe that her real mother was the woman who had abused her. As NIS, she knew better but could not speak the word ‘mother.’

* CEQ-item 7: As a child I often felt lonely: “My mother did not care for me in my childhood, she hit me frequently, and my father abused me. My mother frequently locked me in a cellar. The kettles and the shoeshine box were my best friends.”

* CEQ item 8: As a child I devoted my time to making music, ballet, theater, and/or drawing: ‘I survived thanks to my creativity. My fantasies of living in Australia carried me through my traumatic childhood and adolescence. I secretly expressed my pain in the colors of my embroidery; I had to seek refuge in embroidery because my parents punished me for drawing and painting.’

* CEQ-item 11: Many of my fantasies have a realistic intensity: “Each time my father raped me, I left my body. Looking on the scene, I really believed that not I but some other girl was abused. I still find it very hard to believe that I was that girl.”

* CEQ-item 16: When I recall my childhood, I have very vivid and lively memories: “When I think of my childhood, I am flooded with the idea that I am a very bad person, my parents told me so. Memories of that sometimes become awfully vivid.”

* CEQ-item 18: When I perceive violence on television, I get so into it that I get really upset: A typical response to this item was that observed violence triggers traumatic memories that involve violence. Some also said that they felt an urge to save violated children in films, documentaries, etcetera.

**Discussion**

The present findings are at odds with the idea that patients with DID are generally highly fantasy prone, and that their disorder and memories are due to fantasy gone awry. The mean CEQ score in our sample was lower than the mean CEQ score of amateur actors and actresses, regular visitors of exhibitions about paranormal issues, and fantasy role players, i.e., people who spend a great deal of their free time to re-enact great historical events[5]. These groups had means of 10.8 (SD = 3.9), 11.1 (SD = 4.4), and 13.2 (SD = 4.4), respectively.

The current DID patients also had lower CEQ scores than patients with borderline personality disorder (M=11.8, SD 5.1), a disorder that is not seen as caused by or primarily related to fantasy proneness[7]. Furthermore, the mean CEQ score for the current women with DID was not much higher than the CEQ score for patients with schizophrenia (M=8.4, SD 4.4), and comparable to the level of fantasy proneness in a female high school students, university students, and university employees (M=9.2, SD 4.0). Women with DID are thus generally not highly fantasy prone. In fact, they are comparable to normal women in this regard.

Considering the CEQ scores of patients with DID, it must be noted that some items of the instrument measure common dissociative symptoms in DID, such as ‘feeling controlled by something or someone outside myself’ and out of body experiences. One CEQ item inquires about having felt alone as a child. In fact, many DID patients report that they felt alone as a child in the context of emotional neglect and secretive traumatization. Endorsement of this item may thus be fact-based rather than fantasy-based. Still other items may measure post-traumatic stress symptoms. For example, item 16 can pertain to the reactivation of traumatic memories and item 18 to the reactivation of these memories following confrontation with conditioned trauma-stimuli. The CEQ scores of DID patients were artificially inflated in or sample because of these particular items.

Although DID is not generally associated with high fantasy proneness, future research needs to explore how these patients develop the features of their different dissociative identity states, and the role of imagination and fantasy in these elaborations.

**References**

1. Merckelbach H, Devilly GJ, Rassin E (2002) Alters in dissociative identity disorder. Metaphors or genuine entities? Clin Psychol Rev 22: 481–497.

2. Merckelbach H, Rassin E, Muris P (2000) Dissociation, schizotypy, and fantasy proneness in undergraduate students. J Nerv Ment Dis 188: 428–431.

3. Rassin E, Merckelbach H, Spaan V (2001) When dreams become a royal road to confusion: realistic dreams, dissociation, and fantasy proneness. J Nerv Ment Dis 189: 478–481.

4. Giesbrecht T, Lynn SJ, Lilienfeld SO, Merckelbach H (2008) Cognitive processes in dissociation: an analysis of core theoretical assumptions. Psychol Bull 134: 617–647.

5. Merckelbach H, Horselenberg R, Muris P (2001) The Creative Experiences Questionnaire (CEQ): a brief self-report measure of fantasy proneness. Personality and Individual Differences 31: 987–995.

6. Steinberg M (1993) Structured Clinical Interview for DSM-IV Dissociative Disorders (SCID-D). Washington DC: American Psychiatric Press.

7. Merckelbach H, à Campo J, Hardy S, Giesbrecht T (2005) Dissociation and fantasy proneness in psychiatric patients: a preliminary study. Compr Psychiatry 46: 181–185.
