# Supplementary material for: Fact or Factitious? A Psychobiological Study of Authentic and Simulated Dissociative Identity States
Source: PLoS One. 2012 Jun 29;7(6):e39279. doi: 10.1371/journal.pone.0039279 (PMC3387157; doi:10.1371/journal.pone.0039279)

**Supporting Information S2**

**How well are the dissociative identity disorder simulating healthy controls doing?**

A. A. T. Simone Reinders^1,2^, PhD, Ellert R. S. Nijenhuis^3^, PhD

*^1^ King's College London, Institute of Psychiatry, Department of Psychosis Studies, London, United Kingdom,*

*^2^ Department of Neuroscience, University Medical Center Groningen, and BCN Neuroimaging Center, University of Groningen, Groningen, The Netherlands,*

*^3^ Top Referent Trauma Center Mental Health Care Drenthe, Assen, The Netherlands*

**Introduction**

Did the controls effectively simulate dissociative identity disorder (DID)? That is, did they report the kind of subjective sensori-motor and emotional experiences that the DID patients reported as the neutral identity state (NIS) and trauma-related identity state (TIS)? It might be objected that our main findings (i.e., that there are substantial psychophysiological and neural differences between authentic DID patients and our controls) are due to ineffective simulation of subjectivity in DID rather than to real psychobiological differences between DID patients and controls if this simulation of subjective reactivity to memory scripts would not have been effective. Here we present a factorial statistical analyses of the within group (both high and low fantasy

prone DID simulating controls) subjective reactions (emotional and sensori-motor ratings).

**Methods**

Statistical analyses were performed with SPSS-PC 15.0 (2006) in an identical manner as was done for the

patient data[1,2] and the analyses as reported in the main manuscript. Results with *p* < 0.0083 are corrected for multiple comparisons, results with a *p* < 0.05 are reported as uncorrected significant. Results with a *p* between < 0.05 and < 0.1 are indicated as trends and a *p* of > 0.1 is reported as `n.s.'. Within SPSS a two-by-two factorial design was defined with the first factor being the factor identity state, consisting of the levels NIS and TIS, and the second factor memory script, consisting of the levels neutral and trauma-related. The statistical analyses consist of the two main effects analyses and the accompanying interaction effect. The healthy control data included in the current analyses is identical to the data as included in the analyses in the main manuscript.

**Results**

Table S1 shows that both control groups effectively simulated the subjective reports of the two different identity states of the DID patients. Both the sensory and the emotional ratings were significantly different for the two simulated identity states. The high fantasy prone control group performed slightly better then the

low fantasy prone control group. For the high fantasy prone control group a trend toward statistical significance was found for the sensory rating memory script main effect. The low fantasy prone control groups were performing slightly worse than the high fantasy prone controls. For the low fantasy prone control group a non-significant finding was found for the sensory rating memory script main effect and a trend was found for the sensory rating interaction effect.

Both the sensory and emotional scores are displayed in Figure S1. These figures show that in general the high fantasy prone DID simulating controls were better at simulating the subjective features of the different identity states as compared to the low fantasy prone DID simulating controls.

**Conclusion**

Both high and low fantasy prone DID simulating controls were able to report the subjective emotional reactions of the two different identity states in the DID patients. High fantasy prone DID simulating controls were better at this simulation and both groups had difficulty imitating the reports for sensorimotor reactions to trauma scripts. These results were obtained after brief practise and are in line with the sociocognitive and fantasy-based model of DID. These models predict that subjects with high levels of suggestibility and/or fantasy proneness[3–8] can easily report the subjective reactions of the different identity states in DID. Holders of trauma-related models of DID do not deny that simulation of subjective reports is possible, and it is important to note that subjective reports do not imply subjective experiences.

**References**

1. Reinders AATS, Nijenhuis ERS, Paans AMJ, Korf J, Willemsen ATM, et al. (2003) One brain, two selves. Neuroimage 20: 2119–2125.

2. Reinders AATS, Nijenhuis ERS, Quak J, Korf J, Haaksma J, et al. (2006) Psychobiological characteristics of dissociative identity disorder: a symptom provocation study. Biol Psychiatry 60: 730–740.

3. Rauschenberger SL, Lynn SJ (1995) Fantasy proneness, DSM-III-R axis I psychopathology, and dissociation. J Abnorm Psychol 104: 373–380.

4. Merckelbach H, Rassin E, Muris P (2000) Dissociation, schizotypy, and fantasy proneness in undergraduate students. J Nerv Ment Dis 188: 428–431.

5. Merckelbach H, Horselenberg R, Muris P (2001) The Creative Experiences Questionnaire (CEQ): a brief self-report measure of fantasy proneness. Personality and Individual Differences 31: 987–995.

6. Merckelbach H, Muris P (2001) The causal link between self-reported trauma and dissociation: a critical review. Behav Res Ther 39: 245–254.

7. Giesbrecht T, Merckelbach H (2006) Dreaming to reduce fantasy? – Fantasy proneness, dissociation, and subjective sleep experiences. Personality and Individual Differences 41: 697–706.

8. Giesbrecht T, Merckelbach H, Kater M, Sluis AF (2007) Why dissociation and schizotypy overlap: the joint influence of fantasy proneness, cognitive failures, and childhood trauma. J Nerv Ment Dis 195: 812–818.

** = p < 0.0083 (i.e., corrected for multiple comparisons)

* = p < 0.05 (i.e., uncorrected for multiple comparisons)

DIS = dissociative identity state

MS = memory script

DIS * MS = interaction effect

HRV-AVG = average of normal-to-normal time intervals

**Figure S1:**

Graphical display of the subjective sensori-motor and emotional ratings.


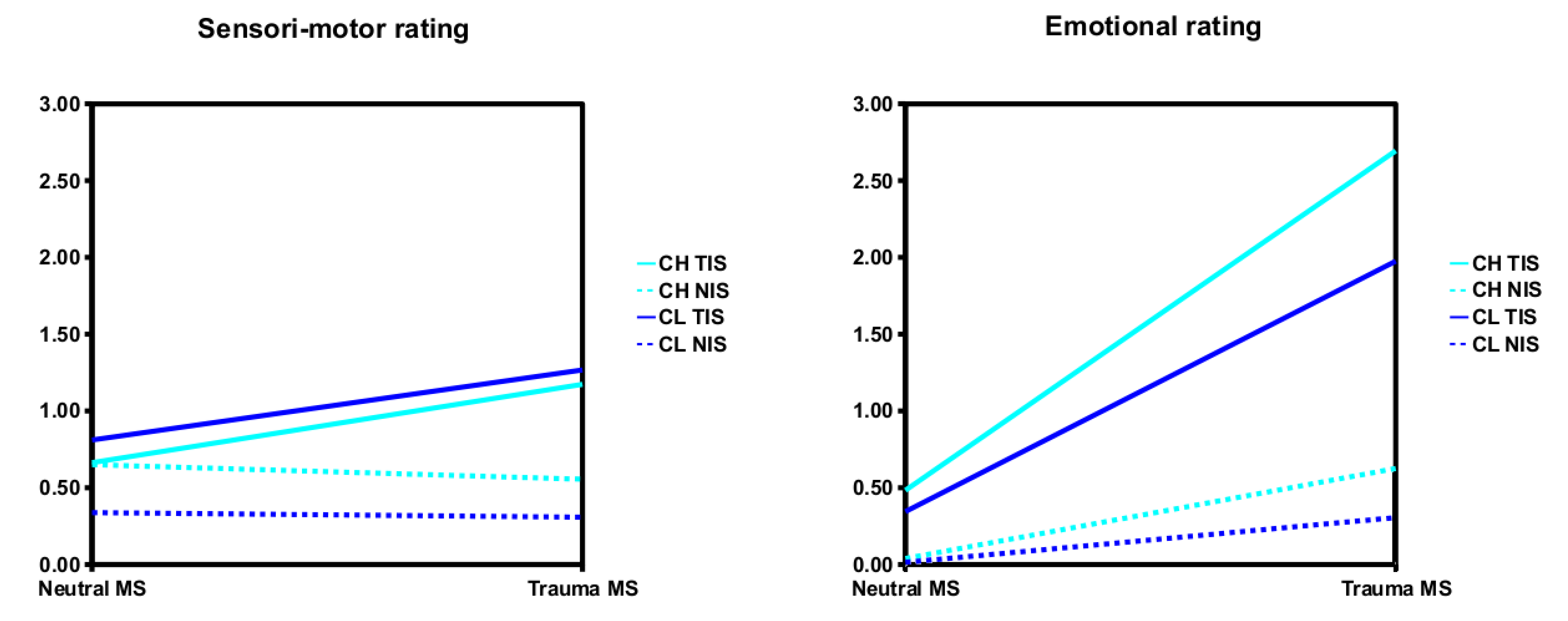

Supplement: Supporting Information S2 — How well are the dissociative identity disorder simulating healthy controls doing? (DOC) [file pone.0039279.s002.doc]
